# Community factors affecting participation in larval source management for malaria control in Chikwawa District, Southern Malawi

**DOI:** 10.1186/s12936-020-03268-8

**Published:** 2020-06-02

**Authors:** Steven Gowelo, Robert S. McCann, Constantianus J. M. Koenraadt, Willem Takken, Henk van den Berg, Lucinda Manda-Taylor

**Affiliations:** 1 Laboratory of Entomology, Wageningen University & Research, Wageningen, The Netherlands; 2 Training and Research Unit of Excellence, School of Public Health, College of Medicine, Blantyre, Malawi; 3 Department of Health Systems and Policy, School of Public Health and Family Medicine, College of Medicine, Blantyre, Malawi; 4 Center for Vaccine Development and Global Health, University of Maryland School of Medicine, Baltimore, MD USA

**Keywords:** Malaria, Larval source management, *Bacillus thuringiensis israelensis*, Community, Malawi

## Abstract

**Background:**

To further reduce malaria, larval source management (LSM) is proposed as a complementary strategy to the existing strategies. LSM has potential to control insecticide resistant, outdoor biting and outdoor resting vectors. Concerns about costs and operational feasibility of implementation of LSM at large scale are among the reasons the strategy is not utilized in many African countries. Involving communities in LSM could increase intervention coverage, reduce costs of implementation and improve sustainability of operations. Community acceptance and participation in community-led LSM depends on a number of factors. These factors were explored under the Majete Malaria Project in Chikwawa district, southern Malawi.

**Methods:**

Separate focus group discussions (FGDs) were conducted with members from the general community (n = 3); health animators (HAs) (n = 3); and LSM committee members (n = 3). In-depth interviews (IDIs) were conducted with community members. Framework analysis was employed to determine the factors contributing to community acceptance and participation in the locally-driven intervention.

**Results:**

Nine FGDs and 24 IDIs were held, involving 87 members of the community. Widespread knowledge of malaria as a health problem, its mode of transmission, mosquito larval habitats and mosquito control was recorded. High awareness of an association between creation of larval habitats and malaria transmission was reported. Perception of LSM as a tool for malaria control was high. The use of a microbial larvicide as a form of LSM was perceived as both safe and effective. However, actual participation in LSM by the different interviewee groups varied. Labour-intensiveness and time requirements of the LSM activities, lack of financial incentives, and concern about health risks when wading in water bodies contributed to lower participation.

**Conclusion:**

Community involvement in LSM increased local awareness of malaria as a health problem, its risk factors and control strategies. However, community participation varied among the respondent groups, with labour and time demands of the activities, and lack of incentives, contributing to reduced participation. Innovative tools that can reduce the labour and time demands could improve community participation in the activities. Further studies are required to investigate the forms and modes of delivery of incentives in operational community-driven LSM interventions.

## Background

In the last decade, remarkable progress has been achieved in the fight against malaria [1]. This is largely attributed to a combination of preventive and curative measures including insecticide-treated bed nets and effective case management [1, 2]. Long-lasting insecticide treated bed nets (LLINs) and indoor residual spraying (IRS) as vector control interventions have made major contributions towards the recent gains [3, 4]. Despite these gains, malaria still remains a major public health problem in Africa as reported by stable or increasing incidence rates over the past few years in many African countries [1]. Development of resistance to drugs [5] and insecticides [6, 7] in the malaria parasites and vectors, respectively, and vector behavioural plasticity, such as outdoor feeding and resting [8], threaten the efficacy of available interventions to reduce the malaria burden.

The shortfalls of the current malaria interventions suggest a need for new strategies that can further reduce malaria transmission. Larval source management (LSM), which controls malaria vector populations through reduced suitability of mosquito larval habitats, is recognized as an effective supplementary tool for malaria control under specific conditions [9, 10]. As a complementary malaria control strategy, LSM could be ideal for situations where vector breeding sites are few, fixed and findable [11]. Other factors cited for adoption of LSM as a complimentary tool include cost-effectiveness when compared with other tools [12, 13] and its ability to control vector populations that avoid contact with insecticide-based tools [14]. Further, the microbial larvicides under advocacy for use in LSM have not, to date, been shown to cause any signs of resistance in vector populations or harmful effects on non-targeted organisms [11]. In Kenya, the deployment of LSM as a complementary measure to communities already using LLINs was shown to significantly improve malaria control compared to the situation with LLINs used as a stand-alone method [9].‬‬‬‬‬‬‬‬‬‬‬‬‬‬‬‬‬‬‬‬‬‬‬‬‬‬‬‬‬‬‬‬‬‬‬‬‬‬‬‬‬‬‬‬‬‬‬‬‬‬‬‬‬‬‬‬‬‬‬‬‬‬‬‬‬‬‬‬‬‬‬‬‬‬‬‬‬‬‬‬‬‬‬‬‬‬‬‬‬‬‬‬‬‬‬‬‬‬‬‬‬‬‬‬‬‬‬‬‬‬‬‬‬‬‬‬‬‬‬‬‬‬‬‬‬‬‬‬‬‬‬‬‬‬‬‬‬‬‬‬ A number of other studies have reported similar results showing the contribution of LSM to malaria reduction in Africa [12, 15–19].

In Malawi, like in many other African countries, LSM has not yet been introduced or evaluated for malaria control. This is due to a number of factors including a lack of data on local larval mosquito vector ecology [20], lack of local evidence for LSM in malaria control, and concerns about the cost of implementation on a large scale. One potential method of managing implementation costs and intervention coverage is to closely involve communities in the application of LSM. This approach could enable adequate coverage of targeted areas through education and skills development of communities about LSM, reduce costs of implementation as human capital is locally available, and increase community acceptance and ownership [21, 22]. A review of case studies concludes that community participation is key to success of interventions [23]. For instance, feasibility of community involvement in LSM has been demonstrated in urban settings in Dar es Salaam, Tanzania, where improved standards of larval surveillance were reported [24].

Participation of communities in malaria control has not been emphasized in Malawi. Instead, community-based management of diseases in hard-to-reach villages has been implemented by government-employed Health Surveillance Assistants (HSAs). Therefore, there is lack of sufficient evidence in the country on whether community engagement in malaria control can increase coverage, acceptance and uptake of control interventions. A five-year community-led malaria control project, Majete Malaria Project (MMP), was implemented in southern Malawi to investigate the additive effect of community participation in malaria control through community workshops on malaria, structural house improvement and LSM on the strategies recommended in the national malaria control policy [25, 26]. The study could contribute to evidence for community engagement in malaria control in Malawi.

The Majete Malaria Project (MMP) was a community-led malaria control project undertaken in villages along the perimeter of the Majete Wildlife Reserve in Chikwawa district in Southern Malawi [25]. Local communities were involved in the development and implementation of the LSM activities as part of MMP [26]. In this study, conducted 2 years after commencement of community involvement in the LSM activities, the factors influencing implementation and acceptability of LSM for malaria control were assessed using a community-driven approach. An understanding of these factors could inform the best practices for future development and deployment of community-based interventions.

## Methods

### Study area

**Fig. 1:**
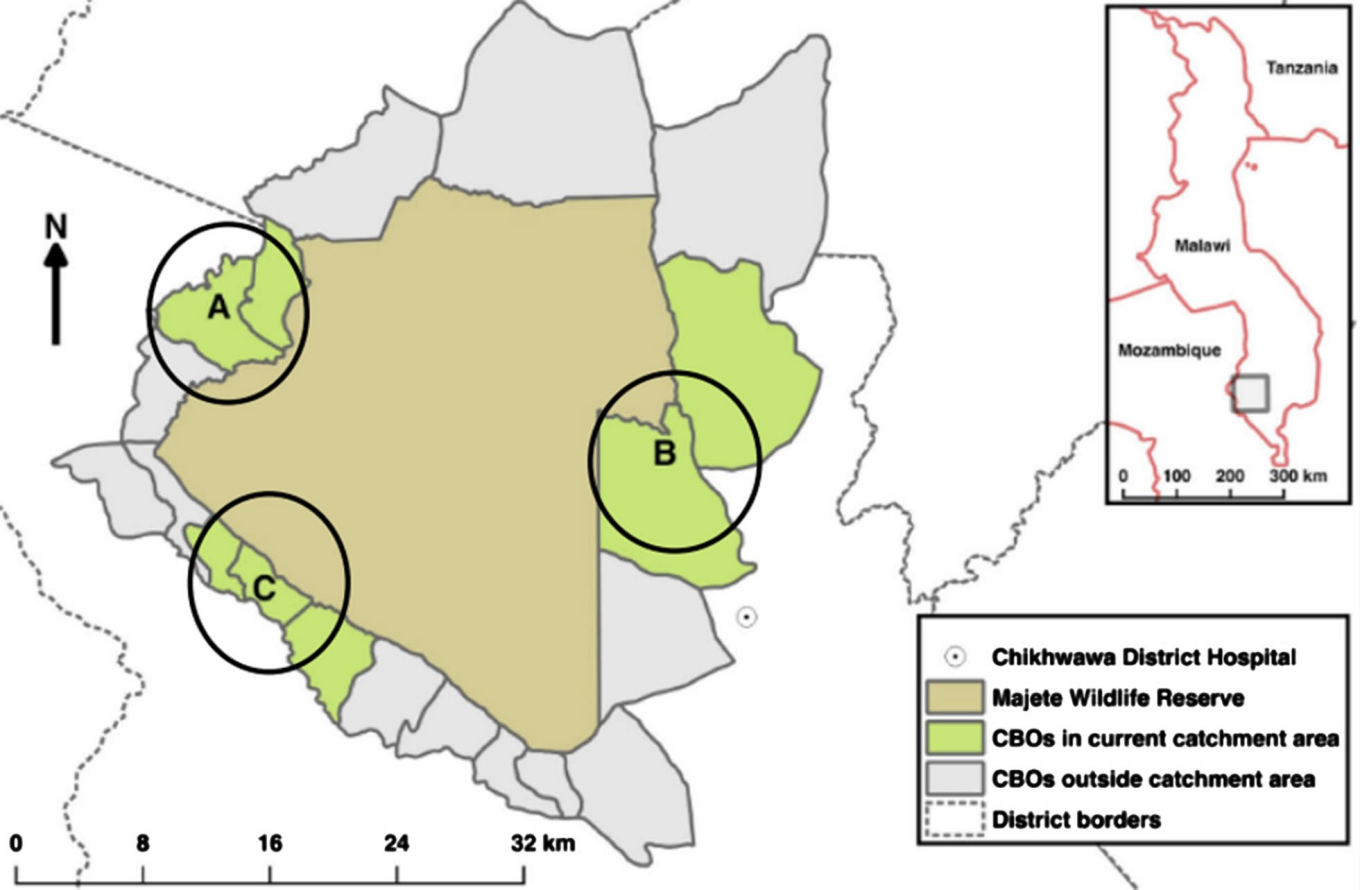
Map of Majete Wildlife Reserve and Majete Perimeter showing the three focal areas

Larval source management was implemented in 26 villages as part of MMP from May 2016 through April 2018 as part of a cluster randomized trial described in detail elsewhere [25, 26]. All 26 villages assigned the LSM arm of the randomized trial were included in the current study. All villages were located along the Majete Wildlife Reserve perimeter in Chikwawa district (16° 1′ S; 34° 47′ E), southern Malawi. Chikwawa is hot and dry from September to December, hot and rainy from January to April, and mild and dry from June to August. The district is generally dry with typical Savannah type of vegetation though agricultural land use is common in the landscape. The majority of people in the study villages keep livestock with cattle, goats and pigs being the predominant animals. Most of the households practice subsistence farming with maize, millet and beans as staple food. The study villages were divided into three sub-regions, called focal areas, spaced roughly evenly around the wildlife reserve and covering a total population of about 25,000 people in 65 villages (Fig. 1) [27].

### Study population

The study was undertaken with community members from the 26 villages spread across the three focal areas, assigned ‘A’, ‘B’ and ‘C’. Three different groups of respondents were identified: (1) health animators (HAs), (2) LSM committee members, and (3) members from the broader community. The HAs and LSM committee members were selected to coordinate the local malaria control initiatives. Selection of these groups was led by was village heads in consultation with members of the community [26]. The HAs and LSM committee members received formal joint training from MMP and The Hunger Project-Malawi (THP) staff on malaria topics, such as vector biology, parasite transmission, and vector control. After training, the HAs were tasked with organizing and conducting village workshops in their respective villages to share knowledge on the malaria topics. They were also responsible for fostering malaria discussions, facilitating community-based implementation of larval habitat draining and filling as part of community-based LSM and coordinating all malaria control activities at village level. The LSM committees were comprised of 10 to 12 individuals from the respective village selected by members of each village at community meetings. These LSM committees were formed to carry out LSM activities in each selected village, and they were tasked with quarterly mapping of potential mosquito larval habitats, lobbying for and coordinating community participation in larval habitat draining and filling, and *Bti* application. Community members were then tasked with larval habitat draining and filling.

### Data collection

Survey instruments comprised of focus group discussions (FGDs) and in-depth interviews (IDIs) that were developed based on points stemming from quantitative surveys conducted by the first author prior to the qualitative study. Prior to commencement of data collection, data collectors were trained and the data collection tools were piloted. This was done in order to acquaint the data collectors with the purpose of the study, interview guides and consent forms, and the consenting process, and data collection using voice recorders. Additional file 1: Table S1 and Additional file 2: Table S2 provide summaries of the interview guides. Questions for the different interview sessions included perception of malaria as a problem, its symptoms, mode of transmission, risk factors and control, and recommendations for effective community involvement in control initiatives. Questions related to the perception of malaria as a problem and knowledge about malaria transmission were restricted to IDIs and FGDs involving the general community.

Twenty-four IDIs were conducted with members from the general community in the study villages. Selection of the IDI participants was based on overall village-level motivation and participation in the LSM activities. This was based on results of the quantitative surveys conducted a priori. To rank the villages, proportions of participants per village who indicated both motivation and participation in the activities were compared with the proportion of those who indicated no or little motivation and participation. Then the villages were divided into two groups: (1) Above average motivation and participation and (2) Below average motivation and participation. Twelve IDIs were conducted with participants from villages with above average motivation and participation, and the other twelve from the villages with below average motivation and participation.

Nine mixed-village FGDs were undertaken with community members, HAs and LSM committee members drawn from different LSM villages. These did not include participants of the IDIs. Like in the IDI sessions, selection of villages from which participants would come was based on how each village ranked on the scale described for the IDIs. Thus, for each mixed-village FGD session the participants came from villages with above average motivation and participation and below average motivation and participation. The FGDs were conducted in each of the three focal areas, such that one FGD for each of the three target groups was conducted in each focal area. To stimulate discussion and ensure contribution of all members the number of participants in the FDGs was between six and eight.

A Consolidated Criteria for Reporting Qualitative Studies (COREQ) highlighting details of methods such as the research team, study design, and analysis and findings has been provided (Additional file 3: Table S3).

### Data analysis

The IDIs and FGDs were conducted in the local language *Chichewa*. All data were audio-recorded, transcribed and translated into English. Data was analysed thematically. The first author familiarized himself with the whole data set and the last author coded four transcripts. A common coding framework was developed through discussion. A codebook was developed using inductive and deductive coding methods. The inductive approach allowed generation of new themes emerging from the data while the deductive approach was based on a pre-developed codebook, which guided the coding process. The translated excerpts were coded using NVivo 12 (QSL international, Victoria, Australia). The first and last author identified key themes.

### Ethical consideration

The University of Malawi’s College of Medicine Research and Ethics Committee granted ethical approval (COMREC protocol number P.12/17/2222). Permission to collect data in the study villages was provided by the Chikwawa District Heath Office (DHO). Prior to recruitment of participants, communication about the study was sent to the community through local village heads in liaison with HAs. Written informed consent was obtained from all participants during data collection. All the participants were men and women aged above 18 years. Literate participants provided a signature on the consent form and illiterate participants provided a thumbprint. Interviews were conducted in a private space, and participants were assured that their personal details would be omitted from transcripts and no personal details would be divulged to ensure confidentiality. Finally, participants were informed that their involvement in the research was voluntary and that withdrawal was permitted at any time and without personal consequence.

## Results

### Respondent characteristics

A total of 87 respondents participated in the 33 interview sessions: 24 IDIs and 9 FGDs (Table 1). All the IDIs were conducted with the community members that were not HAs or LSM committee members. Three FGDs were conducted per focal area: one with community members; one with HAs; and one with LSM committee members. Most of the participants were in the age group 18 to 24 (71.3%) and reported primary education as their highest level of formal education (51.7%). More males (57.5%) than females (42.5%) participated in the interviews.

**Table 1:** Characteristics of study participants

Characteristic	Focal Area (n)			Respondents [N, (%)]
	Focal area A (30)	Focal area B (26)	Focal area C (31)	
Sex				
Female	11	13	13	37 (42.5)
Male	19	13	18	50 (57.5)
Age				
18–24	7	11	5	23 (26.4)
25–44	23	15	24	62 (71.3)
≥ 45			2	2 (2.3)
Education				
None	16	8	2	26 (29.9)
Primary	10	11	24	45 (51.7)
Secondary	4	7	4	15 (17.2)
Tertiary			1	1 (1.2)
Session				
FGD	3	3	3	9 (27.3)
IDI	8	8	8	24 (72.7)

The study results were grouped into five main themes drawn through the inductive and deductive methods (Table 2). Theme 1 covered topics that were only asked to the participants from the general community and not to the other groups (HAs and LSM committee members).

**Table 2:** Main themes drawn from the qualitative study

Theme
Community perception of malaria as a health problem
Community knowledge about malaria transmission
Community trust, support and acceptance of microbial larviciding
Community participation in LSM: enabling and hindering factors
Recommendations for scale-up and future community-led LSM

### Community perception of malaria as a problem

Results of the present study showed widespread perception of malaria as a health problem among members from the broader community. Unlike the HAs and LSM committee members, the broader community received minimal formal training on the malaria topic. Much of their knowledge came from their interactions with the HAs and LSM committee members who received tailored training from the larger project, MMP. The community members mentioned that their own malaria related illness, and/or illness of those close to them, reduced their performance of income generating activities and increased financial expenses via treatment and treatment-seeking activities.

*“When one suffers from malaria they need money to access treatment while at the same time all economic activities that would be undertaken to improve their livelihood are halted”* (IDI, Community participant, Kandeu 2)

*“I find malaria to be particularly burdensome because it is very hard to find medicines at the local health centres hence we are forced to buy from pharmacies at higher prices”* (IDI, Community participant, Kabwatika)

Although malaria was identified as a problem that affects everyone, most participants reported that pregnant women and children are most vulnerable to the disease.

*“Much as everyone is at risk of malaria, young children and pregnant women are the most vulnerable”* (IDI, Community participant, Kampaundi).

### Knowledge of malaria transmission

There was widespread knowledge among all respondents about the mode of transmission of malaria parasites and the type of environment conducive for breeding and development of mosquitoes. Almost all respondents reiterated that bites from infected mosquitoes drive malaria transmission. Interestingly, a member from the community was even able to mention the sex and genus of the mosquito responsible for the transmission of malaria.

*“When a female Anopheles mosquito bites a person with malaria and then another person without the disease, the malaria parasite is transmitted to the latter”* (IDI, Community participant, Chipula).

When asked where mosquito larvae could be found, participants provided varying responses. Some participants identified natural and human-made sites and objects as potential larval habitats. Particular mention was made on the duration of water storage, the nature of the water (stagnant, dirty or clean), including the container or vessel and the contents in it, as factors that contribute to where mosquito larvae are most likely to be found. The term “dirty water” was in these cases synonymous with foul water.

*“Mosquitoes breed in standing water or in water that has been stored or has not been used for a long time”* (FGD, LSM Committee, FA-B)

*“There are some mosquito breeding sites which are natural such as streambeds while many are man*-*made”* (FGD, HA, FA-A)

*“Anopheline larvae are found only in clean water while Culicine larvae are found in dirty water”* (FGD, HA, FA-A)

Most participants implicated human activities with creation of the potential mosquito larval habitats. The responses provided were categorized based on purpose: (1) domestic: washing and drinking, (2) agriculture: irrigation, fish farming and watering points for livestock, (3) and construction: brick-making and mud. Brick-making purposes were the most mentioned reason for creation of larval habitat sites. However, the issue of eliminating these water bodies revealed community perspectives on conflicts between economic activities and malaria control.

As one community member said,

*“These larval habitats came into existence due to development activities being conducted in our communities such as school building initiatives which demand us to make bricks. We are caught in a situation where one development activity affects another” (*IDI, Community participant, Mkangeni)

An LSM committee member who was responsible for carrying out larviciding activities supported this opinion.

*“Much as we know that these are the very places where mosquitoes driving malaria transmission breed, some of these places are very important to us as we use them to irrigate crops, drinking points for our livestock and also to soak bamboos for making traditional mats”* (FGD, LSM committee, FA-A).

Despite the perceived conflict between community developments and malaria control, participants displayed an understanding of the role of these places as refuge for immature stages of mosquitoes. This enabled some of the participants to suggest solutions for malaria control.

*“Mosquitoes breed in standing water bodies which are readily available in our villages. Removing these potential breeding sites is the only sure way forward’’* (IDI, Community participant, Jana)

*“If we are not careful, discharging water anyhow into these swamps creates suitable environments for mosquito proliferation, a thing which can increase malaria prevalence in the area”* (FGD, LSM committee, FAC).

### Community trust, support and acceptance of microbial larviciding

Most of the participants agreed on the effectiveness of *Bti* for mosquito control. However, it was observed that some community members did not want to work with the larvicide for fear of a health risk for themselves or their livestock, especially at the onset of the project. Lack of evidence of the product’s activity and safety was the major reason for the skepticism and lack of trust in the product by the community.

A community member highlights this perception:

*“I do not really know how Bti works but I think it can cause cancer. Because no livestock has died due to the larvicide does not mean I should not be concerned”* (IDI, Community participant, Mkangeni)

Additionally, some participants were initially sceptical about the product, because LSM committee members used mouth masks during application of *Bti*.

*“The use of masks by members of LSM committees during Bti application made some people suspicious of the product”* (FGD, HA, FA-A)

The initial concerns were on the safety of livestock, crops and human life, but as time passed the community members could see that *Bti* did not have harmful effects on their crops, livestock and their personal health. Increased engagement with LSM committees and HAs increased community trust, support and acceptance of the larvicide.

*“Initially we had a lot of fears about Bti as we thought it would be harmful to those using treated water sources but we have neither seen nor heard of any harm due to the larvicide. We are beyond convinced that this product only kills mosquito larvae”* (IDI, Community participant, Kampaundi).

*“We did not allow LSM committees to apply Bti in water bodies, especially those used for irrigations purposes because we had fears the larvicide would cause damage. Now we have realized that our fears were unfounded. We are very willing and ready to have the habitats sprayed with the product”* (FGD, Community members, FA-C)

In some cases, field-based workshops were held with the community where *Bti* was actually applied on habitats infested with mosquito larvae. At these sites the activity of *Bti* on the larvae and other aquatic organisms was co-investigated with the community members.

*“When the intervention just started, people had concerns about harmful effects of Bti on crops, livestock and people. To prove to them that the larvicide was very safe we conducted sensitization meetings in our communities. The communities are now aware that spraying Bti does not introduce any risks to crops, humans and livestock”* (FGD, LSM committee, FA-C)

The LSM committees believed that it was only those people who did not attend community workshops who had negative concerns about the product.

*“The people who complained were those who never attended village workshops so they did not know the benefits of Bti. Once they come to understand they will never protest again”* (FGD, LSM Committee, FA-B)

### Factors enabling participation in LSM activities

Under this theme factors that motivated community participants in carrying out LSM activities were explored. Enabling factors included involvement of local leaders in the initiative and the knowledge gained through workshops about malaria control and implementing control measures. Most LSM committee members felt that the knowledge they attained about mosquito larval control made them aware of their role in the fight against malaria.

*“We have gained a lot of knowledge about the malaria topic from the numerous trainings we have gone through. This knowledge motivates us to participate in the malaria control activities”* (FGD, LSM Committee, FA-B)

*“Our village heads contribute to the cause by organizing community meetings where they encourage us to actively participate in the LSM activities”* (FGD, Community members, FA-B).

The community members perceived a visible decline in malaria cases in their communities, which they attributed to their work. They indicated that such achievements encouraged them to work towards more reductions in the malaria burden. They also cited problems faced to access treatment for malaria as a factor driving their actions towards malaria control.

*“We have had the worst experiences with malaria. We live very far from health facilities hence have problems to access health care services. This initiative is our lifeline hence our great zeal to participate”* (IDI, Community participant, Kampaundi)

*“I am motivated to participate in the activities because our community has been very disadvantaged in terms of access to health care services. We live very far from the nearest health facility, which is also a paying facility. I fully understand the challenges faced to access medical help at the facility. So when we were told about what we are supposed to do to reduce the malaria burden I decided to participate”* (IDI, Community participant, Kandeu 2)

There was a general feeling among the community members that HAs and LSM committee members were more motivated to participate in the LSM activities than the rest of the community. However, the community members expressed mixed sentiments as to why HAs and LSM committees seemed more motivated to participate in the activities. Some community members felt that the knowledge the two groups gained during the course of their duties enticed them to participate in the control initiative. Another section of the community felt that the money given to the two groups by MMP to meet logistical requirements for trainings outside their focal areas incentivised them.

*“These people work hard because they understand that the intervention would be beneficial to their communities”* (IDI, Community participant, Kandeu 2)

*“LSM committee members work hard because they are taken to trainings where they are given money. If there were no such incentives none of them would be as active”* (IDI, Community participant, Kampaundi IDI)

### Factors hindering participation in LSM activities

When asked what they felt were the limiting factors for community implementation of the LSM activities, the respondents cited a number of issues. One of the major factors cited by LSM committee members was the high amount of labour and time required to carry out *Bti* application activities. Weekly applications of *Bti* were necessary for optimum effectiveness of the *Bti* because of its short residual activity. However, LSM committee members reported that much of their time was spent carrying out the LSM activities, which reduced their time to participate in income generating activities for their households.

*“The work is too laborious. We do Bti pre*- *and post*-*spray surveys every week, and we spray Bti after every seven days. This means we spend much of our time working in LSM at the expense of our families’ well*-*being”* (FGD, LSM Committee, FA-C)

They also mentioned the long walking distances to the sites where they applied *Bti* and the continued creation of potential mosquito larval habitats.

*“The major problem is distance, when we go to spray Bti, we travel long distances because some water bodies are very far. Sometimes we plan to spray more breeding sites per day but fail to realize the plan because we have to travel long distances hence end up spraying in very few. This makes us work for more days than expected”* (FGD, LSM Committee, FA-C)

*“This work is very tiresome as we are required to continuously fill and drain, and spray Bti every week in the potential mosquito breeding sites. From the look of things we will continue to create these sites as we do not have alternatives to bricks [the excavation of which creates breeding sites]”* (FGD, LSM committee, FA-A)

Some respondents indicated that provision of no monetary incentives was a major factor influencing lack of participation in the activities. While this feeling was widespread, it was not true for some villages.

*“Some members are discouraged because they want outright benefits. Of course, in my area there have never been such cases, but I know this happens in other villages”* (FGD, LSM Committee, FA-C)

Lack of gumboots as protection from water-borne infections, for example to protect against schistosomiasis, for each committee member was the most cited challenge. While acknowledging the provision of several pairs of gumboots by the project, they noted that these were not sufficient for all committee members. They also indicated their reluctance to share boots due to risk of contracting foot-borne fungal infections.

*“We do not have enough gumboots for all members of the committee. We were told to be sharing the few we have but we cannot do that for fear of athlete’s foot*” (FGD, LSM committee, FA-A)

Some LSM committee members cited the indifference of some community members towards LSM as a demotivating factor. Respondents noted that some community members did not attach value to the work of committee members and demeaned their volunteerism. This indifference left some LSM committee members frustrated, and in some cases led to dropping out from the committees.

*“We are often discouraged by poor remarks from some members of the community despising our volunteerism”* (FGD, LSM committee, FA-B)

*“We are called stupid and time wasters by some community members for volunteering to work in this project”* (FGD, HA, FA-C)

### Recommendations for scale-up and future community-led LSM

There was a widespread perception among the respondents that village heads were not fully involved in the on-going LSM activities. The respondents suggested that for increased community participation in the activities the village heads needed to receive training and be tasked with specific roles. Some participants recommended that for future or for scale-up of existing community-led initiatives, groups comprised of village heads should be created to monitor the activities locally.

*“A team of village heads should be instituted which should be tasked with monitoring LSM activities at village level. They should receive the same training as LSM committees. These people are highly respected by communities, which could ensure high community participation in the LSM activities. This team should be constantly updated by HAs and LSM committees”* (FGD, HA, FA-C).

Some participants also recommended restructuring LSM committees by removing non-active members to improve group performance, adding more members to existing committees to reduce member work-load, or by making the selected committees work for a fixed period after which new committees take over.

*“I think the LSM committees are burdened by the too large amount of work they are doing. It would be a better idea to bring in more people into the committees so the committee can do more sensitization meetings and cover more habitats”* (FGD, Community members, FA-C)

*“I think LSM committees should work for a maximum of one year and a new one be selected. Currently, some members have lost interest in the activities hence need to keep replacing them with new members willing to take over”* (IDI, Community participant, Mkangeni)

The participants also recommended need for constant feedback on how the intervention is progressing. They felt this could encourage their participation in the activities.

*“The community should be given feedback on how the intervention is performing. This could motivate them*” (FGD, HA, FA-A)

Lastly, continued community sensitization was reported to be paramount if buying-in and participation in the LSM activities were to be successful.

*“There is need for continued sensitization meetings. It is through repeated messages that some people change their attitude”* (FGD, HA, FA-A)

## Discussion

Findings of the present study show that community involvement in LSM increased awareness of malaria as a health problem, its risk factors and control strategies. Lack of incentives as observed in other research paradigms in Malawi [28] reduced participation of members from the broader community in the activities. Support from community leaders was a critical factor for community participation in the activities. Labour intensiveness, the time-demanding nature of the activities, and fears about health risks associated with working in water bodies, created barriers to successful implementation of the intervention by the LSM committees. These results suggest that a wide range of factors must be considered for optimum effectiveness of community-driven malaria interventions.

Participants in the present study perceived malaria as a health problem prevalent in their communities and recognized children and pregnant women as groups most vulnerable to the disease. Participants were aware of the role of mosquitoes in transmitting the malaria parasite and had knowledge of potential mosquito larval habitats. This knowledge is attributable to the malaria workshops conducted by the HAs in each village. Previous studies have suggested that community awareness of malaria as a burden has the potential to trigger positive action towards malaria control [29, 30].

‬‬‬‬‬‬‬‬‬‬‬‬‬‬‬‬‬‬‬‬‬‬‬‬‬‬‬‬‬At the time of the present study, the results of the intervention trial [25] were not yet available, and hence in the preparatory meetings with communities, as well as during the refresher courses for Health Animators and local leaders, participants were reminded of the possible outcomes: no impact on malaria, medium impact on malaria or strong impact on malaria. Local communities needed to be encouraged to continue with the interventions which in the end might benefit them.

In this study, the communities understood the association between mosquito larval habitats and malaria. However, some water bodies served a specific function in the community and were deemed useful by the respondents. This presents potential limitations in the adoption of habitat draining and filling for malaria control. Similar observations were made in Kenya where perceived importance by the community of some water bodies limited their willingness to remove such sites [31]. Where habitat draining and filling are not feasible, application of larvicides is a viable alternative [9], and this was widely practiced by the communities in this study. ‬‬‬‬‬‬‬‬‬‬‬‬‬‬‬‬‬‬‬‬‬‬‬‬‬‬‬‬‬‬‬‬‬‬‬‬‬‬‬‬‬‬‬‬‬‬‬‬‬‬‬‬‬‬‬‬‬‬‬‬‬‬‬‬‬‬‬‬The use of other LSM strategies such as predatory fish or shading of the breeding sites with plants such as Napier grass or coco-yams to make such sites less suitable for malaria vector mosquitoes has also been suggested [32].

Community perception of *Bti* as a mosquito control tool improved with increased engagements with HAs and LSM committees, and interaction with the product. Initially, the communities reported skepticism about the product over potential harmful effects to humans, livestock and crops. The lack of a befitting synonym for the word “pesticide” when referring to *Bti* in the participants’ vernacular, *Chichewa*, confounded their fears of the product. In *Chichewa*, the word “pesticide” is loosely interpreted as “poison” which denotes an inherent element of side effects. Through community workshops and handling of the product in the field, the community learned about the product’s activity and specificity, which resulted in improved acceptance of the product by the community. Similar observations were made in Rwanda where acceptance of *Bti* was observed to improve with increased interaction with the product by rice farmers tasked with its application [33]. The findings suggest that for meaningful acceptance of control strategies, community training should focus on approaches that build trust by demonstrating the safety of the products to non-target organisms.

The HAs and LSM committees were more motivated to participate in the LSM activities than the members from the community at large. According to the HAs and LSM committees, attainment of knowledge of malaria and its control, and their sense of ‘duty’ motivated their participation in the LSM activities. For the both groups, the status received in the community for their role made them feel valued and motivated. However, some members from the broader community felt that the motivation of the HAs and LSM committees was a result of the “monetary incentives” they received during their trainings. This could be justified by the frequent calls made by the LSM committees for refresher trainings. This could potentially pose a barrier in community participation in the intervention as observed in another study conducted in Malawi where receipt of incentives by some groups demotivated other groups [34]. Similarly, in a sub-study conducted under MMP in the same area as the current study “monetary incentives’’ received by the HAs during their trainings were feared to have weakened the sustainability of the health animator approach [35]. Indeed, the forms and modes of delivery of incentives in volunteer-based initiatives are critical but they remain less studied [36]. In Kenya, adaptation of a malaria control intervention (odour-baited mosquito traps) to local context by providing a source of solar energy to householders increased community acceptance and uptake of the intervention [37]. Based on these findings, incentives have a role in influencing acceptability, uptake and sustainability of community-led interventions. To increase interest of a community and motivation to participate, the intervention agenda should be developed in light of the local contexts, with enhanced attention for the community’s needs.

Participants considered LSM activities to be labour intensive and time consuming, especially larviciding with *Bti*, which required weekly application. Some LSM committee members felt that the demands of the activities prevented them from actively engaging in income generating activities for the betterment of their livelihoods. The findings underscore the need to incorporate technical solutions that increase intervention coverage and quality while reducing labour demands. These technical solutions include powered sprayers, drones, and remote-sensing based risk maps [38, 39].

In the present study, it was evident that local leadership was needed for effective implementation of the community-led LSM activities. A hierarchical structure with village heads, HAs and LSM committees as leaders was regarded as supportive by most of the respondents. This finding suggests that local authorities should not be engaged for administrative purposes only but also in both planning and implementation of community-led initiatives. The findings also suggest that the village heads should work closely with LSM committees and HAs, with the latter groups only addressing the operational aspects and not the village politics such as calling for community workshops. Importantly, interventions should capitalize on the existing traditional structures present in each community. Rural communities have strong social structures resulting from their communal living [31] which, if exploited, could make community engagements attainable.

The variable participation of members from the broader community reported in the present study likely resulted in lower coverage of LSM than was targeted. Though not measureable in the absence of intervention because the FGDs did not include an external control group, the community’s high level of knowledge about malaria as a health problem and its transmission is presumably due to the educational intervention of MMP. This suggests that the conception and design of the intervention were effective.

## Conclusions

Community involvement in LSM as an additional tool for malaria control increased local awareness of malaria as a health problem, its risk factors and control strategies. However, community participation varied among the respondent groups, with labour and time demands of the activities, and lack of financial incentives, among the reasons cited for reducing participation. Employing innovative tools with potential to reduce labour and time demands could improve community participation in the activities. Further studies are required to investigate the forms and modes of delivery of incentives in operational community-driven LSM interventions.

## Supplementary information

**Additional file 1: Table S1.** Interview guide (FGD) for Community members, HAs and LSM committees.

**Additional file 2: Table S2.** Interview guide (IDI) for Community members.

**Additional file 3: Table S3.** A *COREQ* checklist highlighting details of methods.

## Data Availability

Data can be made available upon reasonable request.
