# Supplementary material for: Community factors affecting participation in larval source management for malaria control in Chikwawa District, Southern Malawi
Source: Malar J. 2020 Jun 2;19:195. doi: 10.1186/s12936-020-03268-8 (PMC7265157; doi:10.1186/s12936-020-03268-8)
Supplement: Supplementary file 2 — Additional file 2: Table S2. Interview guide (IDI) for Community members. [file 12936_2020_3268_MOESM2_ESM.docx]

**Additional file 2: Table S2:** Interview guide (IDI) for Community members.

| 1. What are the most common health problems in this community? |
| --- |
| 1. Is malaria considered a serious health problem in this community? Why? |
| 1. Who do you perceive to be the most susceptible to malaria? |
| 1. In your opinion how do people get malaria? |
| 1. Do you think it is possible to control mosquitoes? Explain your response |
| 1. What kind of things do people in this community usually do to protect themselves from malaria? |
| 1. Do you practice (some of) these preventive measures? Which measures do you practice? |
| 1. What do you know about mosquito larval control? |
| 1. Who do you think should take the lead in mosquito larval control? Why? |
| 1. How would you describe your roles as a community member in malaria control? |
| 1. What are the common mosquito breeding sites in your area? Probe: How did these water bodies come into existence? Of what importance are they to communities? How would you relate presence of these water bodies with malaria transmission? |
| 1. What do you know about mosquito larval control using *Bti*? Probe: where would you recommend use of *Bti* as opposed to other larval control initiatives i.e. draining and filling? |
| 1. How are the LSM activities implemented in your village? Probe: Do you think the activities are too laborious or not? If yes, what factors make the activities demanding? How would you suggest the activities be approached? |
| 1. What challenges do you encounter as you carry out the activities? How do you deal with each challenge? |
| 1. What factors make some members of the committee not to actively participate in the LSM activities? |
| 1. For those actively participating in the activities, what reasons could be attributed to their motivation? |
| 1. What is the community's perceptions about *Bti*? What are the comments generally made about the intervention? |
| 1. What changes, if any, have you seen in terms of malaria cases since the Majete Malaria Project came into your community? |
| 1. What do you think should be done to improve community participation in community-led malaria control? |
