# Supplementary material for: Community factors affecting participation in larval source management for malaria control in Chikwawa District, Southern Malawi
Source: Malar J. 2020 Jun 2;19:195. doi: 10.1186/s12936-020-03268-8 (PMC7265157; doi:10.1186/s12936-020-03268-8)
Supplement: Supplementary file 3 — Additional file 3: Table S3. A COREQ checklist highlighting details of methods. [file 12936_2020_3268_MOESM3_ESM.docx]

**Additional file 3: Table S3:** A COREQ checklist highlighting details of methods.

| **No** | **Item** | **Description** |
| --- | --- | --- |
| **Domain 1: Research team and reflexivity** |  |  |
| Personal Characteristics |  |  |
| 1 | Interviewer/facilitator | All interviews were conducted by 9 trained interviewers. |
| 2 | Credentials | Each interviewer possessed at least a university first degree. |
| 3 | Occupation | All the interviewers were recruited from a list of data collectors involved in other sub-studies under MMP done *a priori* |
| 4 | Gender | Eight interviewers were male, one was female |
| 5 | Experience and training | 1. Prior to commencement of data collection, interviewers were trained and the data collection tools were piloted. 2. The data collectors had prior experience in qualitative data collection. |
|  |  |  |
| Relationship with participants |  |  |
| 6 | Relationship established | Few participants had a previously established relationship with the interviewers (based on prior data collections conducted in the same study area) |
|  |  |  |
| 7 | Participant knowledge of the interviewer | A minority of the participants knew the interviewers. |
| 8 | Interviewer characteristics | It was reported to the participants that the interviewers were recruited independent of the larger project, MMP, therefore, no bias was created. |
| **Domain 2: study design** |  |  |
| Theoretical framework |  |  |
| 9 | Methodological orientation and Theory | Thematic framework analysis |
| Participant selection |  |  |
| 10 | Sampling | Purposive [methods] |
| 11 | Method of approach | Recruitment involved face-to-face invitations following prior communication about the study to the community through local village heads in liaison with HAs |
|  |  |  |
| 12 | Sample size | Eighty-seven [methods and results] |
| 13 | Non-participation | All residents approached gave consent and participated in the study |
| Setting |  |  |
| 14 | Setting of data collection | The data was collected at participants’ homes or at freely agreed locations at the participant’s convenience . |
| 15 | Presence of non- participants | No non-participants were present during data collection |
| 16 | Description of sample | Twenty-three participants were between 18 and 24 years old while the remaining 64 were at least 25 years old, 50 of the participants were male and 26 of the participants received no formal education. [results] |
|  |  |  |
| Data collection |  |  |
| 17 | Interview guide | The interview guide is appended to the manuscript. |
| 18 | Repeat interviews | We did not carry out any repeat |
| 19 | Audio/visual recording | All interviews were audio recorded and the recordings were transcribed. [methods] |
|  |  |  |
| 20 | Field notes | Field notes were made during the interview and later summarized. |
| 21 | Duration | Each interview lasted 40-60 minutes |
| 22 | Data saturation | Sampling was continued until sufficiency was achieved |
| 23 | Transcripts returned | We did not return transcripts to participants for comment or correction because they were anonymized at the time of transcription |
|  |  |  |
| **Domain 3: analysis and findings** |  |  |
| Data analysis |  |  |
| 24 | Number of data coders | All transcripts were coded by Steven Gowelo; however, Lucinda Manda-Taylor read a sample of transcripts and provided analytical insights that informed coding. |
|  |  |  |
| 25 | Description of the coding tree | We did not provide a description of the coding tree. Only the main theme are provided in this paper. |
| 26 | Derivation of themes | A codebook was developed using inductive and deductive coding methods for derivation of themes [methods] |
| 27 | Software | NVivo |
| 28 | Participant checking | We did not perform member checking |
| Reporting |  |  |
| 29 | Quotations presented | Participant quotations are provided in the results to illustrate themes. |
| 30 | Data and findings consistent | To ensure consistency of data and findings the last and first authors cross-checked other's codebook. |
|  |  |  |
| 31 | Clarity of major themes | Our results present the major themes, Table 2. |
| 32 | Clarity of minor themes | Minor themes are discussed under each major theme. |
